# MRI, PET/CT and PET/MRI Fusion in the Assessment of Lymph Node Metastases in Head and Neck Cancer

**DOI:** 10.3390/diagnostics16020252

**Published:** 2026-01-13

**Authors:** Nikolaus Poier-Fabian, Christian Asel, Hanna Cristurean, Michael Mayrhofer, Veronika Moser, Jan Maximilian Janssen, Thomas Ziegler, Michael Gabriel, Nina Rubicz, Paul Martin Zwittag

**Affiliations:** 1 Department of Otorhinolaryngology, Head and Neck Surgery, Kepler University Hospital GmbH, Krankenhausstrasse 9, 4021 Linz, Austria; nikolaus.poier-fabian@kepleruniklinikum.at (N.P.-F.); paulmartin.zwittag@kepleruniklinikum.at (P.M.Z.); 2 Medical Faculty, Johannes Kepler University Linz, Altenberger Strasse 69, 4040 Linz, Austria; 3 Department of Radiology, Kepler University Hospital GmbH, Krankenhausstrasse 9, 4021 Linz, Austria; 4 Institute of Anatomy and Cell Biology, Medical Faculty, Johannes Kepler University Linz, Krankenhausstrasse 5, 4020 Linz, Austria; 5 Institute of Nuclear Medicine and Endocrinology, Kepler University Hospital GmbH, Krankenhausstrasse 9, 4021 Linz, Austria

**Keywords:** MRI, PET/CT, PET/MRI fusion, cervical lymph nodes, lymph node metastasis, head and neck cancer

## Abstract

**Background/Objective:** The aim of the present study is to compare diagnostic accuracies of MRI, PET/CT and fused PET/MRI in the assessment of cervical lymph nodes in patients with head and neck cancer (HNC). **Methods:** Imaging data of 37 patients who underwent MRI, PET/CT, and surgery at our center were retrospectively merged into PET/MR images. Histopathological results of neck dissections and lymph node resections served as the gold standard. **Results:** MRI and PET/CT were performed on the same day. The mean interval between imaging and surgery was 20 (±19.5) days. All three imaging modalities identified the same number of true positive and false negative cases, resulting in identical sensitivity estimates of 66.7%. Specificities were 90.9% for MRI, 95.5% for PET/CT, and 100% for PET/MRI. The corresponding positive predictive values (PPVs) were 83.3%, 80.7%, and 81.5%, while the negative predictive values (NPVs) were 80.0%, 90.9%, and 100%, respectively. Ten false results are further analyzed regarding side and level of the affected lymph node, and intersections of the three modalities are displayed. In 12 (32.4%) cases, additional findings are depicted in PET/CT, 5 (13.5%) of which are histologically confirmed to be further malignancies. **Conclusions:** Software-based PET/MRI is an easy-to-perform procedure and provides valuable clinical information in select clinical questions. Furthermore, whole-body acquisition by PET/CT leads to a notable number of additional malignant diagnoses, which especially favors its use in high-risk patients.

## 1. Introduction

Imaging of head and neck cancer (HNC) is a difficulty clinicians face in everyday practice. In particular, the complex anatomy of this small area, as well as a multitude of possible pathologies, presents a significant diagnostic challenge [1]. Nodal staging (N-staging), at the same time, is essential for treatment planning and prognosis in head and neck squamous cell carcinoma (HNSCC) [2]. Positive N-staging reduces the expected survival rate by approximately 50%, especially in HPV-/p16-negative tumors [3]. At initial consultation, approximately two thirds of the patients with HNC present with locally advanced (LA) disease, including either a large tumor extension and/or the presence of lymph node metastasis (LNM) [4,5,6]. The number of pathologically positive lymph nodes indicates a reduced outcome in patients with HNC. Therefore, accurate identification and characterization of LNM by non-invasive imaging has critical prognostic significance [5,7,8,9,10]. From a surgeon’s point of view, accurate information about the presence and location of LNMs is important in planning appropriate surgery. Surgeons want to address all involved levels while at the same time avoiding unnecessary extension of surgery [10,11,12]. Since extensive neck dissection increases the risk of perioperative blood transfusion and postoperative functional limitation, medical imaging plays a major role in detecting LNMs [2,13].

Cross-sectional imaging modalities like MRI do not solely rely on size and morphological criteria in the assessment of lymph nodes. Since metastasis can be present in normal-sized lymph nodes and enlarged lymph nodes can be benign, advanced functional MRI methods are used to improve detection of LNMs. This includes diffusion and perfusion imaging methods as well as contrast-enhanced MRI [5,14,15,16,17]. Metastasis may be associated with increased cell density, leading to altered water diffusivity that can be measured using diffusion-weighted (DWI) MRI [5]. Dynamic contrast-enhanced (DCE) MRI, on the other hand, is used to assess tumor vascularity. Ultimately, a variety of different characteristics, alone or in combination, can lead to a suspicious finding in MRI [14,17].

Multimodal imaging using positron emission tomography/computed tomography (PET/CT) has gained wide acceptance as a reliable imaging tool for the assessment of lymph nodes, as well as for assessing advanced and recurrent disease [6,18]. ^18^F-fluorodeoxyglucose (FDG) is a glucose analog preferentially taken up by cells with high glucose utilization, a feature of most cancer cells. According to various authors, PET/CT using FDG seems to be superior to CT and MRI in the detection of locoregional nodal and distant metastases [6,7,19,20].

In the evaluation of HNC, many different imaging modalities are applicable. As each commonly used technique, such as ultrasound, CT, MRI and PET, has significant specific inherent weaknesses, a combination is often used in the initial staging of HNC [21]. Due to the excellent soft-tissue discrimination capabilities of MRI and the functional implications of FDG-PET, a combination of these modalities can be advantageous, especially in the evaluation of neck LNs [6,22].

The purpose of this study is to compare the diagnostic accuracy of MRI, FDG-PET/CT and retrospectively fused FDG-PET/MRI in the assessment of cervical lymph nodes in patients with HNC. Sensitivities, specificities, as well as positive and negative predictive values are provided for all modalities using the histopathological results of surgical specimens as the gold standard. Finally, we aim to outline clinical implications and generate further research hypotheses.

## 2. Patients and Methods

### 2.1. Study Design and Population

This retrospective, single-center study includes thirty-seven patients who underwent MRI of the head and neck plus FDG-PET/CT. After image acquisition, all patients received either diagnostic cervical lymph node resection or therapeutic neck dissection at Kepler University Hospital Linz between December 2011 and March 2020. Classical HNC or adenoid cystic carcinoma was noted in all patients. Imaging was performed either as primary staging, after diagnosis and before therapy, or as secondary staging after therapy in the follow-up of other malignancies. Additional findings in PET/CT are defined as results that led to further investigation. Patients with distant metastasis (stage IVc), for whom surgery was contraindicated, are not included in the study.

The study was approved by the ethics committee of the Federal State of Upper Austria and was conducted in accordance with the Declaration of Helsinki (EK No. 1249/2021). Due to the observational and retrospective nature of the present study, the requirement for informed consent was waived by the ethics committee. All investigators have full access to the dataset used for this analysis.

### 2.2. Data Collection, Image Interpretation and Gold Standard

Information about the patients’ sex, age on the day of surgery, diagnosis, imaging results, operative details and histological findings were retrospectively extracted from patient records. Data collection was conducted in accordance with the regulations on data protection. Imaging results were taken from the medical reports. Every case with discordant or unclear results in MRI, PET/CT or histology was reviewed by an experienced nuclear medicine specialist (MG–PET/CT) or a radiologist (CA). PET/CT images were reviewed by MG, and MR images were reviewed by CA. The reviews took place without knowledge of histopathological results or reported findings in other imaging methods. The merged PET/MR images were interpreted by an experienced nuclear medicine specialist (MG) without knowledge of the patient’s history or other imaging results. Histopathological results served as the gold standard for the performance evaluation of the imaging methods. Follow-up data was collected for descriptive purposes only, and was not taken into account for diagnostic accuracy evaluation of pre-therapeutic imaging. The localization of every suspect lymph node in imaging, as well as each pathologically confirmed lymph node in histology, is classified according to the Neck Dissection Classification proposed by Robbins [23].

### 2.3. Image Acquisition

MRI and PET/CT imaging are performed as primary staging, or during post-therapeutic follow-up of other malignancies. In all cases, MRI and PET/CT are performed on the same day, and 1.5-Tesla scanners(Siemens Healthineers, Erlangen, Germany) are used for MRI examinations. T1-turbo spin echo (TSE), short-tau inversion recovery (STIR) and dynamic contrast-enhanced (DCE) sequences are commonly performed in axial and coronal orientation. Diffusion-weighted (DWI) sequences are partially added in axial orientation.

PET/CT scans are performed using a Siemens Biograph 40 Truepoint PET/CT scanner (Siemens Medical Solutions, Hoffman Estates, IL, USA). To ensure blood glucose levels below 150 mg/dL, blood samples are obtained after at least 6 h of fasting and before the injection of ^18^F-fluorodeoxyglucose (FDG). FDG administration takes place 60 min before imaging at a dose of 3.7 MBq/kg (0.0001 Ci/kg). Overlapping sequential emission scans of the whole body are acquired at 3 min per bed position in 3D mode using the axial field of the CT scan.

### 2.4. Image Fusion

Imaging data of MRI and PET/CT are transferred to a Hermes Workstation (Hermes Medical Solutions, Stockholm, Sweden). An automated algorithm based on reciprocal information is used for the anatomical co-registration, as implemented in the Hybrid Viewer (release version 2.18) software package. This automatic process utilizes the anatomical correspondences of the CT and MRI datasets. Spatial transformation of the CT scan is applied for optimized alignment. Every image fusion is reviewed by a radiology technologist and small adjustments are applied if necessary. Contrast-enhanced (DCE) T1-TSE sequences are used for image fusion.

### 2.5. Surgery

**Figure 1:**
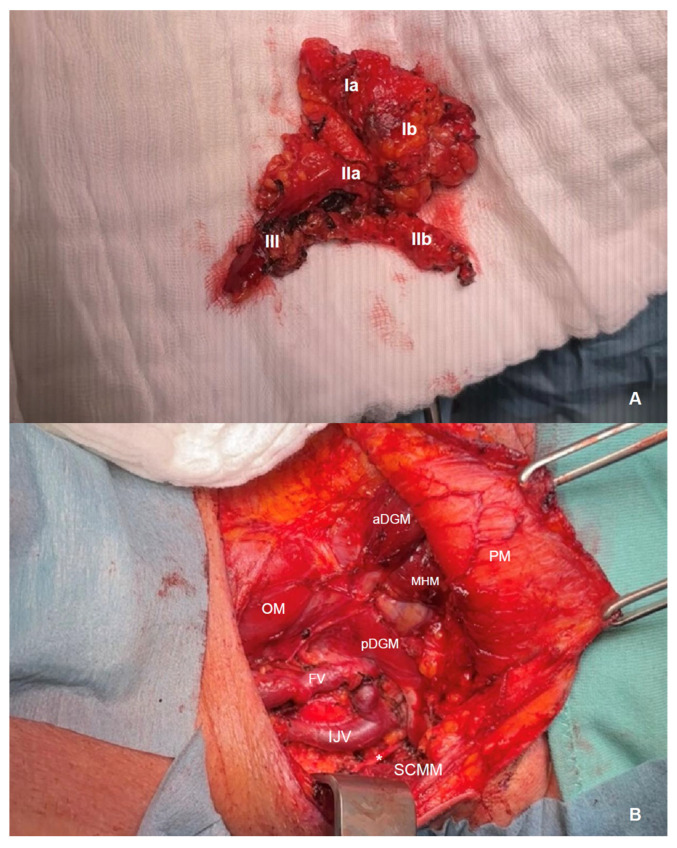
Exemplary intraoperative images of a neck dissection of the left side in a patient with carcinoma of the oral cavity in our center. (**A**) En bloc resected neck dissection preparation with adipose tissue, lymph nodes and lymph vessels of levels I–III (Ia+b, IIa+b and III) including the upper part of the facial vein and the submandibular gland (Ib). (**B**) Neck situs from the same perspective after resection [a+pDGM anterior and posterior belly of the digastric muscle, PM platysma muscle, MHM mylohyoid muscle, OM omohyoid muscle, FV facial vein (resected in its upper part), IJV internal jugular vein, * accessory nerve (eleventh cranial nerve, CN XI), SCMM sternocleidomastoid muscle].

Uni- or bilateral neck dissection is performed in the majority of cases as part of the therapeutic concept of HNC (see Figure 1). Surgery followed a standardized head and neck surgery approach, when pre-therapeutic imaging showed negative neck lymph nodes. It was adapted according to imaging results when positive nodes appeared in unexpected neck levels. Diagnostic lymph node resection alone is performed in only a very limited subset of cases. Surgery is performed after image acquisition in all patients and the histological result is considered as the gold standard.

Frequencies of tumor stages are counted for all cases of HNC and adenoid cystic carcinomas. Elevated risk of lymph node metastasis is defined as pathologic T classification ≥ 3, lymphatic invasion, grading ≥ 3 and/or p16 positivity.

### 2.6. Follow-Up

Since 2017, patients with HNC treated at our center have undergone a follow-up regime of 5 years. A baseline staging is performed 3 months after the end of therapy with head and neck MRI and a CT scan of the thorax and upper abdomen. During the first 2 years, a clinical evaluation is performed every 3 months, whereas in the following 3 years, clinical evaluation is performed in 6-month intervals. MRI is repeated annually in all patients, beginning one year after diagnosis. In cases with advanced cancer stages or additional risk factors, annual CT imaging of thorax and upper abdomen is also performed.

The duration of follow-up in the study group is presented in months (±SD). Follow-up ends when local relapse or distant metastasis is detected, or after 5 years for patients treated after October 2017. Patients treated prior to 2017 remain in lifelong follow-up.

### 2.7. Statistical Analysis

Data is entered into Microsoft Excel and then transferred to SPSS Statistics 25.0 (IBM Corp., Armonk, NY, USA). Descriptive statistics are calculated to assess and show frequencies. Nominal variables are displayed in absolute and relative frequencies, whereas metric variables are presented with mean values and standard deviation (SD). Cross-tables are used to show sensitivity, specificity, positive predictive value (PPV) and negative predictive value (NPV) with confidence intervals (CI). The McNemar test is used to analyze dichotomous variables and the Stuart–Maxwell test is performed for multinomial variables. A *p*-value < 0.05 is considered statistically significant.

## 3. Results

### 3.1. Patient Characteristics

Patient characteristics, imaging, malignancies, surgeries and follow-up, as well as time from imaging to surgery are shown in Table 1. Each category is presented for the entire cohort and stratified by sex.

Thirty-seven patients had a mean age of 61.9 (±12.4) years on the day of surgery. Ten (27.3%) are female and 27 (73.9%) are male. Thirty-five (94.6%) patients had head and neck squamous cell carcinoma (HN-SCC) and two (5.4%) patients had adenoid cystic carcinoma. Thirteen (35.1%) patients were staged as stage I, eight (21.6%) as stage II, nine (24.3%) as stage III, six (16.2%) as stage IVa and one (2.7%) patient as stage IVb. Stage IVc patients were not included due to inherent contraindication for surgical treatment. Twenty (55.6%) patients have elevated risk of lymph node metastasis in histology of the primary site. Fifteen (40.5%) patients have pathologically confirmed lymph node involvement. In two (5.4%) patients, imaging is performed as secondary staging during follow-up for other malignancies. PET/CT detected additional suspicious findings that led to further investigation in 12 (32.4%) patients. Bilateral neck dissection (ND) was performed in 23 (62.2%), unilateral ND in 12 (32.4%) and lymph node resection alone in 2 (5.4%) cases. The mean time from imaging to surgery is 20 ± 19.5 days. The mean time of follow-up is 59.6 ± 30.9 months. None of the patients developed distant metastasis within the first year after surgery (see Table 1).

### 3.2. Diagnostic Accuracy

Cervical lymph node malignancy was confirmed by histology in 15 (40.5%) patients. Imaging with MRI, PET/CT and PET/MRI detected suspicious lesions in the neck in 12 (32.4%), 11 (29.7%) and 10 (27.0%) patients, respectively.

The sensitivity and specificity of MRI are 66.7% (CI 38.4–88.2%) and 90.9% (CI 70.8–98.9%) and the PPV and NPV are 83.3% and 80.0%, respectively. PET/CT shows a sensitivity and specificity of 66.7% (CI 38.4–88.2%) and 95.5% (CI 77.2–99.9%), respectively, with a PPV and NPV of 80.7% and 90.9%.

PET/MRI fusion yields a sensitivity and specificity of 66.7% (CI 38.4–88.2%) and 100% (CI 88.4–100%), respectively. PPV and NPV are 81.5% and 100%, respectively. McNemar’s test does not show any significant differences for MRI vs. histology (*p* = 0.453), PET/CT vs. histology (*p* = 0.219) or PET/MRI vs. histology (*p* = 0.63).

### 3.3. Intersection of False Results

**Figure 2:**
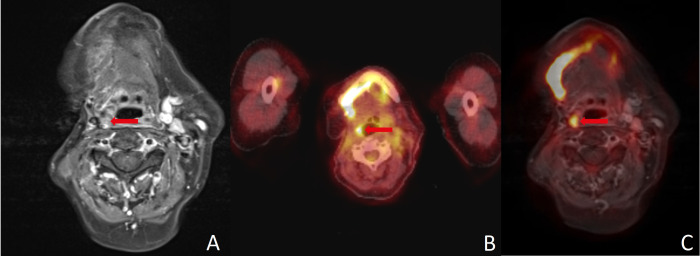
(**A**) MRI yielded a false negative finding, whereas (**B**) PET/CT, (**C**) PET/MRI and histopathology all confirmed malignancy. The FDG uptake combined with high soft-tissue contrast in (**C**) PET/MRI can simplify image interpretation and help guide surgery. Red arrows point to the region of interest in all three imaging modalities.

Patients with malignancy confirmed by histology were assessed as false negative in five (13.5%) cases by MRI, in five (13.5%) cases by PET/CT and in five (13.5%) cases by PET/MRI (see Figure 2). In one (2.7%) case, both MRI and PET/CT were false negative and PET/MRI identified a suspicious lymph node. Conversely, one (2.7%) patient was diagnosed false negative in PET/MRI, while MRI and PET/CT detected a suspicious finding (see Figure 2).

**Figure 3:**
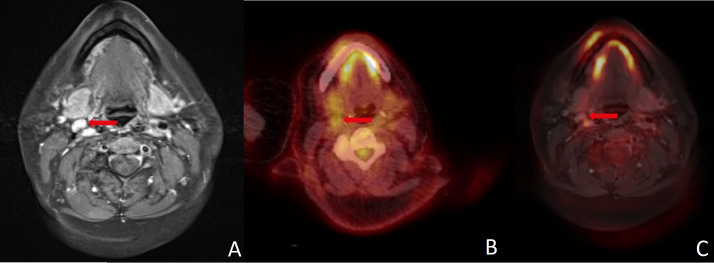
(**A**) Size and contrast enhancement in MRI were misleading in this histopathologically negative neck. Absence of FDG uptake led to correct negative results in (**B**) PET/CT and (**C**) PET/MRI. A combination of different modalities, image fusion in particular, can improve the assessment of cervical lymph nodes. Red arrows point to the region of interest in all three imaging modalities.

MRI indicated LNM in two (5.4%) histologically negative patients. PET/CT was false positive in one (2.7%) patient and PET/MR did not depict any false positive neck findings (see Figure 3). In seven (18.9%) patients with divergent results between imaging methods, one did not show an elevated risk of LNM in histology.

### 3.4. Side and Location of Malignancy

With false negative and false positive cases excluded, the side of malignancy was classified correctly in all unilateral cases by MRI. In one case, MRI, PET/CT and PET/MRI described suspect lesions in both sides of the neck, although malignancy was confirmed for one side only by histology. The Stuart–Maxwell test yielded *p*-values of 0.659, 0.900 and 0.016 for MRI, PET/CT and PET/MRI, respectively. Due to the low number of subjects, a statistically significant difference cannot be demonstrated.

In the assessment of the level of LNM, one patient with metastasis confined to level II was classified by MRI to have metastases at multiple levels. PET/CT, excluding false negative and false positive cases, describes the correct location of metastases. PET/MRI classified one patient with metastases in multiple levels as having metastasis at level II only, and one case of level II metastasis with suspicious lymph nodes at multiple levels.

### 3.5. Additional Findings in PET Scan

**Figure 4:**
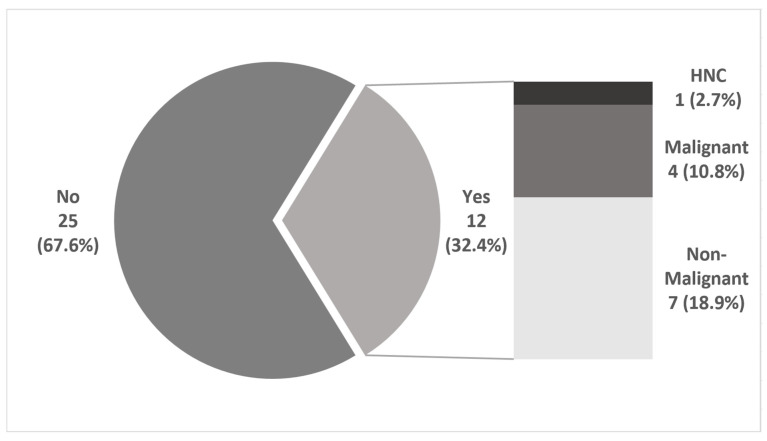
Additional findings in PET/CT. In 25 (67.6%) patients, there was no suspect lesion described in PET/CT besides HNC. In 12 (32.4%) cases with a new diagnosis of HNC, PET/CT showed an additional finding that led to further investigation, four (10.8%) of which were synchronous malignancies. In one (2.7%) case, PET/CT was performed as secondary staging of another malignant disease and HNC was the additional finding.

In 12 (32.4%) patients, PET scans showed additional lesions that led to further investigation. In five (13.5%) patients, further malignant diseases were verified. In four (10.8%) cases, synchronous lung cancer was confirmed histologically. In one (2.7%) patient, HNC was detected incidentally when PET scanning was performed as secondary staging for another malignancy (see Figure 4).

### 3.6. Follow-Up

The mean period of follow-up was 59.6 ± 30.9 months. None of the patients developed distant metastasis within the first year after surgery. Three patients were lost to follow-up during the same period for different reasons. One (2.7%) patient received the follow-up elsewhere, one (2.7%) patient died within a few days after surgery and one (2.7%) patient was lost to follow-up for unknown reasons.

## 4. Discussion

Detection of cervical lymph node metastases in patients with head and neck cancer is crucial. Locoregional LNMs have significant implications both for therapy and prognosis of these malignant diseases. Accurate assessment of the locoregional extent is also important for the surgeon, since the results determine the scope of surgery and adjuvant therapy [6,18,24,25]. The presence of LNM can up-stage HNC and significantly worsen the prognosis of the patient [6].

Both MRI and FDG-PET/CT have proven to have good diagnostic accuracy for detecting LNM in HNC in previous investigations [6,17,26,27,28,29]. Studies investigating the simultaneous use of MRI and PET have shown promising results in terms of diagnostic accuracy for detecting cervical LNMs [5,6,30,31]. To determine the clinical value of PET/MRI fusion, we compared the results of MRI, PET/CT and PET/MRI in patients with HNC. All patients received MRI and PET/CT on the same day and surgery afterwards. Histopathological results served as the gold standard. MR images and PET data were merged retrospectively for research purposes only. To the best of our knowledge, this study is the first to investigate the diagnostic accuracy of retrospectively fused PET/MRI in a cohort of this size at a single center. The results are consistent with existing data regarding the diagnostic accuracy of MRI and PET/CT. From a clinical perspective, a reduction in false negative results is particularly desirable [6]. Unfortunately, data from the present investigation did not demonstrate evidence in this direction for any of the examined modalities. Although PET/MRI did not show any false positive results in our data, false negatives were as high as in MRI and PET/CT. In our cohort, at least one modality showed a false negative result in seven (18.9%) patients. In this context, a combination of modalities could be beneficial as there were only two (5.4%) patients where all modalities showed a false negative result. However, we do not suggest omitting neck dissection based solely on imaging findings. Nonetheless, in selected cases with high intraoperative risk, a combination of different imaging modalities is favorable for guiding therapeutic decisions (see Figure 3).

A major limitation of nearly all imaging methods is the detection of small LNMs. Yamazaki et al. found true positive LNMs in PET/CT to have a mean diameter of 13.4 mm, whereas false negatives had a mean diameter of only 3.1 mm [32]. Accordingly, they described the diagnostic accuracy to be poor in nodes smaller than 10 mm [32]. The challenge in this respect is that a large proportion of LNMs are smaller than this margin [33]. Since MRI attempts to overcome this limitation using DWI and DCE, it appears promising to combine PET with DCE-MRI. However, our data did not provide evidence for improved assessment of small nodes, although in one case FDG-PET could reveal LNM in a positive neck that was not registered in MRI (see Figure 2). Interestingly, elevated risk of LNM in histology (T classification ≥ 3, lymphatic invasion, grading ≥ 3 and/or p16 positivity) seems to be associated with a higher risk of divergent results in different imaging modalities, since only one of seven patients with divergent imaging results did not have elevated risk in histology.

**Figure 5:**
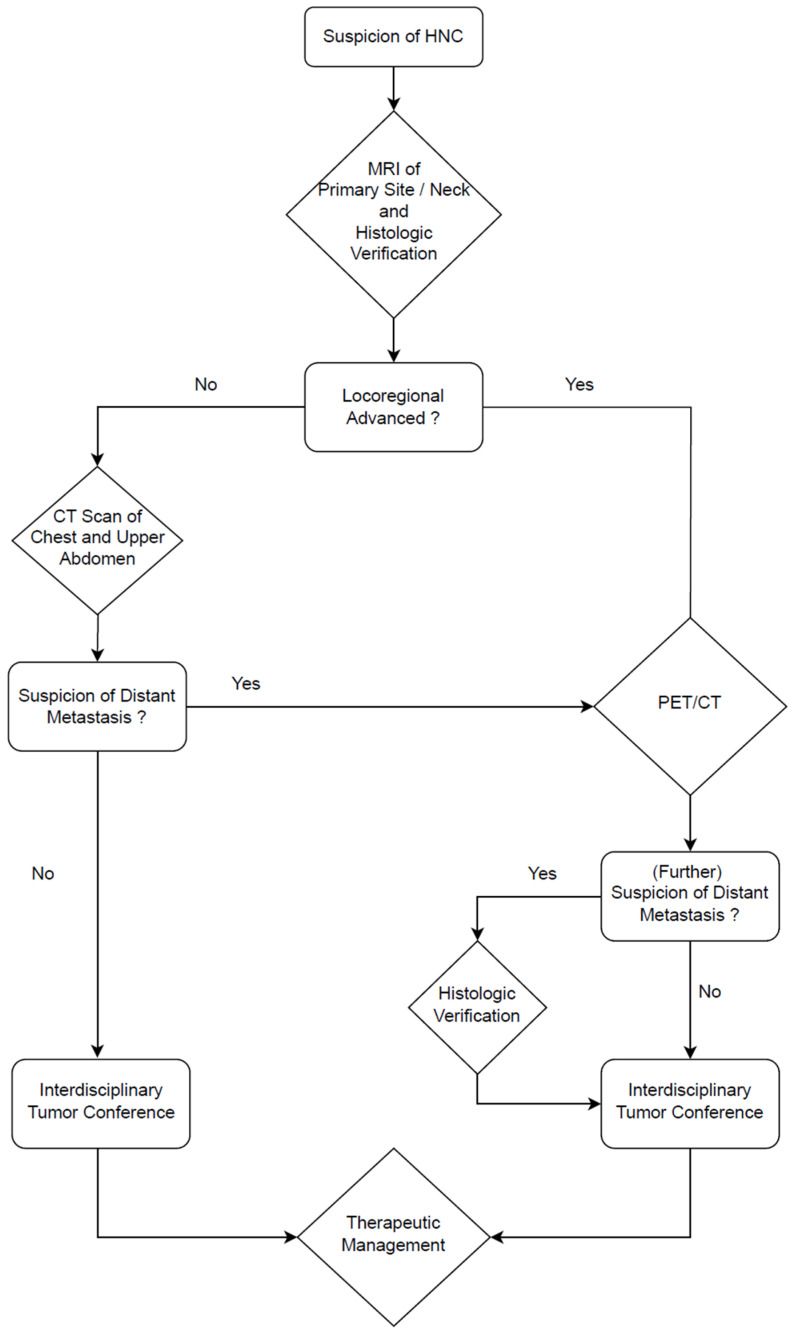
Diagnostic algorithm for HNC workup at our center (modified from Zwittag et al. [6]). DCE-MRI, as described in Section 2.3, is performed in every patient to evaluate the primary site and the neck. In early stages, staging is completed with a CT scan of the chest and upper abdomen. In advanced stages, PET/CT is performed to assess for distant metastases or additional malignancies. An interdisciplinary tumor conference is held for all cases before treatment.

Another important aspect in the workup of patients with HNC is M-staging. Although in our study, patients with distant metastasis were excluded because stage IVc cancer is a contraindication for surgery, there is data indicating the benefit of a whole-body PET/CT workup (see Figure 4). Our data reveal that a remarkable proportion of nearly one third with additional findings leads to further investigation. Notably, additional malignant disease was found in four (10.8%) patients. In one (2.7%) further case, HNC was the additional finding in the secondary staging of another malignancy. Taking this into consideration, the authors recommend a broader use of PET/CT in the workup of HNC, not only for recurrent but also in locally advanced HNC (see Figure 5).

Recent studies have demonstrated a significant impact of immune checkpoint inhibitors on disease-free survival (DFS) when used perioperatively in patients with LA HNSCC [25,34]. One study demonstrated that the postoperative addition of nivolumab to the standard-of-care (SOC) adjuvant chemoradiotherapy increased the 36-month DFS from 52.5% to 63.1% [25]. Another study found that neoadjuvant plus adjuvant administration of pembrolizumab to the SOC therapy (with or without chemotherapy) in resectable LA HNSCC resulted in an increased 36-month DFS from 44.6–46.4% to 57.6–59.8%, depending on the combined positive score (CPS) [34]. This is noteworthy for two reasons in the context of this study. Firstly, since LNMs are defining for LA HNSCC, this underscores the importance of diagnostic accuracy in neck lymph node assessment for further therapy decisions. Secondly, previous work from our group suggests major potential for PET/CT, especially for pre-immune checkpoint inhibitor therapy assessment to stratify patients for maximal therapeutic benefit [35]. Therefore, this upcoming major change in the therapeutic management of HNC is a promising field for further investigation of the functional aspects of FDG-PET/CT and FDG-PET/MRI in HNC diagnostics.

In summary, our data demonstrate comparatively good diagnostic values for all three methods, MRI, alone, PET/CT and merged PET/MRI. The authors maintain that PET/MRI fusion can be beneficial in special cases or peculiar clinical questions, as well as for research purposes. Still, our data do not point out a significant benefit from the fusion of FDG-PET and MRI in the daily routine workup of patients with HNC.

### Limitations

There are several limitations to note. One is the relatively small cohort included in this study. To the best of our knowledge, no other study to date has investigated PET/MRI fusion for the assessment of LNMs of HNC in a larger cohort. Another limitation is that the results of MRI and PET/CT were interpreted non-blinded, introducing the potential for cross-modality bias. However, we attempted to reduce this bias by choosing only cases where the image acquisition was performed on the very same day. PET/MRI, on the other hand, was interpreted in a blinded manner. A general limitation, in this respect, is the retrospective character of the PET/MRI fusion and its interpretation in our analysis, compared to MRI and PET/CT, which were evaluated before therapeutic procedures. Another limitation is that the results of MRI and PET/CT are based on non-blinded findings, potentially leading to the possibility that the results of MRI and PET/CT could influence each other. However, we attempted to reduce this bias by choosing only cases where the image acquisition was performed on the very same day. PET/MRI, on the other hand, was interpreted in a blinded manner. Another general limitation of PET/MRI is the current lack of specialists proficient in interpreting both MRI and PET scans, which may affect diagnostic accuracy. This last point, however, allows for potential improvement of the diagnostic accuracy of PET/MRI with more common use in the future. An inherent limitation of PET/MRI fusion is the fact that fusion of different imaging modalities can lead to mismatches, despite the use of state-of-the-art software by highly experienced technologists.

## 5. Conclusions

In the present study, we evaluated the diagnostic accuracy of MRI, PET/CT and fused PET/MRI in the assessment of cervical lymph nodes in patients with HNC. All three modalities demonstrated comparable diagnostic performance, with PET/MRI showing similar sensitivity to MRI and PET/CT and perfect specificity in our patient group. In our cohort, only 2 of 15 patients with histologically confirmed malignancy had false negative findings across all three modalities. In contrast, using any single modality would have missed five of these cases, indicating that a multimodal approach could potentially reduce false negative results by approximately 60%. Furthermore, whole-body PET/CT acquisition leads to a notable number of additional findings, including a high proportion of further malignancies. Due to the retrospective design and small cohort size, PET/MRI fusion cannot yet be recommended for routine clinical use, but it is an easy-to-perform procedure that provides clinically valuable information in selected cases. Larger prospective studies are needed to confirm these findings and clarify the potential role of PET/MRI in clinical decision-making. Furthermore, we suggest a comparative analysis of simultaneous PET/MRI.

## Figures and Tables

**Table 1:** Demographics, tumor and imaging characteristics and follow-up.

*n* = 120	Female	Male	Overall
Sex	10 (27.03%)	27 (73.9%)	37 (100%)
Age in years (mean)	72.3 ± 12.8	58.1 ± 9.8	61.9 ± 12.4
Diagnosis			37 (100%)
Squamous Cell Carcinoma	8 (80%)	27 (100%)	35 (94.6%)
Adenoid Cystic Carcinoma	2 (20%)	0 (0%)	2 (5.4%)
Tumor stage			37 (100%)
Stage I	5 (50.0%)	8 (29.6%)	13 (35.1%)
Stage II	1 (10.0%)	7 (25.9%)	8 (21.6%)
Stage III	3 (30.0%)	6 (22.2%)	9 (24.3%)
Stage IVa	1 (10.0%)	5 (18.5%)	6 (16.2%)
Stage IVb	0 (0%)	1 (3.7%)	1 (2.7%)
Stage IVc	0 (0%)	0 (0%)	0 (0%)
Elevated risk of LNM in histology			37 (100%)
No	4 (40.0%)	12 (44.4%)	16 (43.2%)
Yes	6 (60.0%)	15 (55.6%)	21 (56.8%)
Lymph node involvement			37 (100%)
No	5 (50%)	17 (63%)	22 (59.5%)
Yes	5 (50%)	10 (37%)	15 (40.5%)
Staging			37 (100%)
primary	8 (80%)	27 (100%)	35 (94.6%)
secondary	2 (20%)	0 (0%)	2 (5.4%)
Additional finding PET/CT			37 (100%)
No	5 (50%)	20 (74.1%)	25 (67.6%)
Yes	5 (50%)	7 (25.9%)	12 (32.4%)
Surgery			37 (100%)
ND bilateral	3 (30.0%)	20 (74.1.6%)	23 (62.2%)
ND unilateral	5 (50.0%)	7 (25.9%)	12 (32.4%)
Lymph node resection	2 (20%)	0 (0%)	2 (5.4%)
Time MRI to surgery (d)	17.7 (±14.3)	19.9 (±19.5)	20 (±19.5)
Time PET/CT to surgery (d)	17.7 (±14.3)	19.9 (±19.5)	20 (±19.5)
Metastasis at follow-up (1y)			34 (100%)
No	9 (100%)	25 (100%)	34 (100%)
Yes	0 (0%)	0 (0%)	0 (0%)
Follow-up time in months (mean)	48.8 ± 30.3	63.5 ± 30.3	59.6 ± 30.9

## Data Availability

The original contributions presented in this study are included in the article. Further inquiries can be directed to the corresponding author.
